# Exploring weight bias and negative self-evaluation in patients with mood disorders: insights from the BodyTalk Project

**DOI:** 10.3389/fpsyt.2024.1407474

**Published:** 2024-05-30

**Authors:** Paolo Meneguzzo, Simone C. Behrens, Chiara Pavan, Tommaso Toffanin, M. Alejandra Quiros-Ramirez, Michael J. Black, Katrin E. Giel, Elena Tenconi, Angela Favaro

**Affiliations:** ^1^Department of Neuroscience, University of Padova, Padova, Italy; ^2^Padova Neuroscience Center, University of Padova, Padova, Italy; ^3^Department of Psychosomatic Medicine and Psychotherapy, Medical University Hospital Tübingen, Tübingen, Germany; ^4^Max Planck Institute for Intelligent Systems, Tübingen, Germany; ^5^Department of Medicine, University of Padova, Padova, Italy; ^6^Psychiatric Unit, Padua University Hospital, Padova, Italy; ^7^Department of Neuroscience and Rehabilitation, University of Ferrara, Ferrara, Italy; ^8^Psychology Department, University of Konstanz, Konstanz, Germany

**Keywords:** weight bias, affective disorder, depression, body weight, implicit bias

## Abstract

**Background:**

Negative body image and adverse body self-evaluation represent key psychological constructs within the realm of weight bias (WB), potentially intertwined with the negative self-evaluation characteristic of depressive symptomatology. Although WB encapsulates an implicit form of self-critical assessment, its exploration among people with mood disorders (MD) has been under-investigated. Our primary goal is to comprehensively assess both explicit and implicit WB, seeking to reveal specific dimensions that could interconnect with the symptoms of MDs.

**Methods:**

A cohort comprising 25 MD patients and 35 demographically matched healthy peers (with 83% female representation) participated in a series of tasks designed to evaluate the congruence between various computer-generated body representations and a spectrum of descriptive adjectives. Our analysis delved into multiple facets of body image evaluation, scrutinizing the associations between different body sizes and emotionally charged adjectives (e.g., active, apple-shaped, attractive).

**Results:**

No discernible differences emerged concerning body dissatisfaction or the correspondence of different body sizes with varying adjectives. Interestingly, MD patients exhibited a markedly higher tendency to overestimate their body weight (p = 0.011). Explicit WB did not show significant variance between the two groups, but MD participants demonstrated a notable implicit WB within a specific weight rating task for BMI between 18.5 and 25 kg/m^2^ (p = 0.012).

**Conclusions:**

Despite the striking similarities in the assessment of participants’ body weight, our investigation revealed an implicit WB among individuals grappling with MD. This bias potentially assumes a role in fostering self-directed negative evaluations, shedding light on a previously unexplored facet of the interplay between WB and mood disorders.

## Background

Mood disorders (MD) represent a complex and significant group of psychiatric conditions that profoundly impact emotional states. These encompass diagnostic categories such as bipolar and depressive disorders (1). From a cognitive point of view, MDs involve self-oriented negative thoughts characterized by selective attention to internal or external stimuli, repetitive thinking, and biased reasoning processes (2). These cognitive patterns are observed in both unipolar and bipolar disorders (3, 4), making them critical targets for cognitive treatments (2).

From a psychopathological point of view, this negative thinking is related to poor self-esteem, affecting mental and physical health (5), with specific implications for depressive psychopathology (6). Self-esteem and self-perception contribute to depression and body dissatisfaction, suggesting a role for cognitive patterns in the development and maintenance of depression symptoms (5, 7). The link between depression and body evaluation often involves negative perceptions and judgments of one’s body and has been stressed particularly in the context of eating disorders and body dysmorphic disorder (8). However, it seems simplistic to focus only on this population due to the existing knowledge that weight bias (WB) seems to persist as one of the few remaining forms of discrimination that society tolerates (9).

WB encompasses a spectrum of negative attitudes, beliefs, judgments, and stereotypes directed toward individuals based on their body weight, including both explicit and implicit biases. It extends beyond literal weight measurements to incorporate societal perceptions of body shape and size, exerting a profound influence on body evaluation (10, 11). WB may significantly influence the mood spectrum due to internalized self-stigmatization (12) and may manifest in various ways, including overt discrimination, subtle microaggressions, and internalized self-stigmatization, contributing to psychological distress. Explicit biases are conscious and intentional, while implicit biases operate at an unconscious level (13). Evaluating both conscious attitudes and unconscious biases allows us to capture a more comprehensive understanding of individuals’ perspectives and behaviors, providing valuable insights for targeted interventions and contributing to a broader societal shift in perceptions, due to the presence of different cognitive information processing systems (14). WB plays a crucial role in self-evaluation and exhibits intricate relationships with psychopathological expressions such as disordered eating, body dissatisfaction, and body shape overvaluation (15–17). Negative self-evaluation stemming from WB contributes to psychological distress, potentially distorting self-schema, and contributing to conditions such as eating disorders and overweight (10, 18, 19). This bias could also explain the high comorbidity rates between depression, eating disorders, and weight fluctuations (20–22). Psychiatric and medical disorders often correlate with different aspects of body-related judgments, such as weight-related attitudes, internalized thinness ideals, and fear of weight gain (17). Longitudinal studies emphasize the impact of poor body image on psychopathological trajectories, influencing well-being, anxiety, and depression (23–25).

A single study has examined the connection between depressive symptoms and body size misperception, revealing that greater clinical symptoms correlate with increased perceptual body image and increased body dissatisfaction (26), prompting further discussion. Internalization of WB predicts poorer mental and physical health, with positive body feelings that counteract internalized weight stigma and encourage healthy behaviors (27, 28). However, existing studies often focus on nonclinical populations (29), and the relationship between depression and WB remains debatable, with evidence supporting bidirectional influences (30–32). Recent reviews confirm a robust association between depression severity and body weight dissatisfaction, even in limited studies of mood spectrum disorder (26). However, the high weight prevalence in most participants limits generalizability (33), highlighting the need for more research on WB, especially among clinically depressed individuals. For these reasons, we decided to apply the methods used in the field of weight and eating disorders to individuals with mood disorders. Indeed, several aspects have been identified as shared pathophysiological mechanisms that may involve eating dysregulation, mood dysregulation, impulsivity, and compulsivity (34). This calls for further studies to enhance our understanding of the clinical overlap between these two conditions (35).

The BodyTalk Project endeavors to comprehensively evaluate implicit and explicit body image perceptions within diverse populations afflicted by eating and weight disorders (18, 19), employing a semantic methodology to unravel the linguistic portrayal of bodies within clinical settings. A pivotal component of this project involves the utilization of realistic, metrically accurate 3D human avatars, serving as a visual representation of body stereotypes by correlating perceived 3D body shapes with linguistic descriptions of body shapes (36, 37). This approach facilitated a deeper understanding of how language influences perceptions of body image, offering valuable insights into the intricate interplay between language and body representation in clinical contexts.

Addressing the similarities in cognitive vulnerabilities between major depression and bipolar disorder (4) and their potential links with negative self-judgment (38), our study aims to expand the knowledge of weight-related dysfunctional cognitive biases in MD patients, potentially informing interventions. This exploratory pilot analysis examines the representation of mental body image in MD patients, with a focus on implicit and explicit weight biases. Our primary hypothesis posits a self-related WB due to underlying cognitive self-judgments in people with MD compared to the general population. Additionally, our objective is to specifically investigate body image disturbance, a less explored aspect in MDs.

## Methods

### Participants

Patients diagnosed with a MD were recruited from the outpatient services of the Psychiatric Clinic of the University Hospital of Padova. Control participants were drawn from the local community and consisted of willing volunteers who participated in a body evaluation study. The specific exploration of WB remained undisclosed until the completion of the computerized task. A total of 25 MD patients (including 20 women) and 35 comparison peers (CPs, with 30 women) were included in the study, carefully matched for sex and age, as detailed in the Results section.

Experienced clinicians conducted clinical diagnoses and collected data, adhering to the criteria outlined in DSM-5. Diagnoses comprised a spectrum of bipolar disorder (10 out of 25 patients) and major depressive disorder, confirmed using the research version of the structured clinical interview for DSM-5 (SCID-5) before starting the task (39). To be eligible for study participation, patients needed to report sustained stability of mood symptoms for a minimum of six months, indicated by the absence of medication modifications or hospitalizations. Both MD patients and their comparison peers were excluded if they had a history of eating disorders. In addition, for CPs, stringent exclusion criteria included a history of psychiatric or medical conditions, neurological trauma, or disorders.

All participants, who were adults, gave their informed consent before engaging in the study. The research adhered to the ethical principles of the Declaration of Helsinki and received approval from the local ethics committee. Comparison peers were recruited from the general population, participated without any form of compensation, and were reached through public announcements distributed through social media.

### Procedure

After enrolling, participants were instructed to schedule a session for the research task, during which they were informed about the study’s focus on body image. Before engaging in computerized tasks, participants were asked to complete self-reported demographic and other questionnaires. These questionnaires were presented anonymously, without full names, ensuring participant anonymity. Subsequently, the participants completed two computerized tasks designed to assess WB and body representation. To mitigate potential carry-over effects, the sequence of tasks and the materials employed remained consistent between all participants.

The entirety of the experiment encompassed approximately 30 minutes and was conducted face-to-face, with participants utilizing a 17” laptop, within a tranquil environment.

### Study materials

Before participating in the assigned tasks, demographic details were systematically gathered. This comprehensive information covered variables such as height, weight, age, years of education, and the duration of their respective disorders.

The evaluation of various psychopathological constructs involved the use of well-established and validated self-report questionnaires. To gauge explicit WB, the Fat Phobia Scale (FPS) was administered. Meanwhile, implicit WB was evaluated by implementing a dedicated computer task (elaborated below).

### Assessment of self-report of psychopathology and body image

The comprehensive psychopathological assessment encompassed a battery of four self-report questionnaires.

The Patient Health Questionnaire (PHQ-9): A 9-item self-report questionnaire utilized to gauge depression within both clinical and general populations (40). Each item investigates the presence of a DSM criterion for a depressive episode in the past two weeks. Responses are structured on a Likert scale with four gradations: 0 (“not at all”), 1 (“few days”), 2 (“more than half the days”), and 3 (“almost every day”). Higher scores correspond to elevated depressive symptoms. The internal consistency for this study was excellent (Cronbach’s α = 0.88).

The Rosenberg Self-Esteem Scale (R-SES): Comprising 10 items, this self-report questionnaire evaluates self-esteem (41). Responses are ranked on a 4-point Likert scale, spanning from “strongly agree” to “strongly disagree.” Higher scores indicate increased self-esteem. The reliability of the scale in this study was robust (Cronbach’s α = 0.91).

The Drive for Thinness (DT) and Body Dissatisfaction (BD) Scales (from the Eating Disorders Inventory): these scales, encompassing 16 items, delve into specific dimensions of body assessment (42). Responses are gauged on a 6-point Likert scale. Internal consistency was found to be satisfactory, with Cronbach’s α values of 0.81 and 0.90 for DT and BD, respectively.

The Fat Phobia Scale (FPS): Comprising 14 items, this self-report inventory targets the explicit WB construct (43). Following a literature-based approach, participants are asked to imagine a specific individual described by two adjectives: “worker” and “obese” (44, 45). Participants then rank these adjectives on a scale from 1 to 5 based on how well they correspond to their feelings and beliefs (e.g., “no will power” *vs*. “will power”). The internal consistency for this scale was sound (Cronbach’s α = 0.79).

### Computerized assessment of weight bias and body representation

To probe implicit WB, we employed two computerized tasks specifically designed to assess the cognitive representation of diverse body shapes (18). Our approach adopted a semantic framework to evaluate both body shapes and WB, employing a set of 16 adjectives carefully chosen from the prior literature (36). These adjectives included descriptors such as active, apple-shaped, attractive, clumsy, determined, feminine, heavy-set, hourglass-shaped, impulsive, insecure, lazy, open-minded, pear-shaped, smart, thin, and unfriendly.

The bodies featured in these tasks were sourced from a previous study, which used a statistical model based on real human body scans to generate a data set of images (36, 46). Each participant was presented with bodies of the same gender as the one with which the participant identified.

Task 1: Rating task. The initial task, referred to as the “rating task”, encompassed 12 images depicting prototypic bodies spanning various weights, coupled with the set of adjectives. Careful selection ensured the inclusion of bodies across a spectrum of BMI values, ranging from 15.5 to 36.5 kg/m^2^. Participants were instructed to assess the applicability of each adjective to the various bodies using a 4-point scale, which ranged from “very much” to “not at all”. The presentation of both adjectives and body images was randomized.

**Figure 1:**
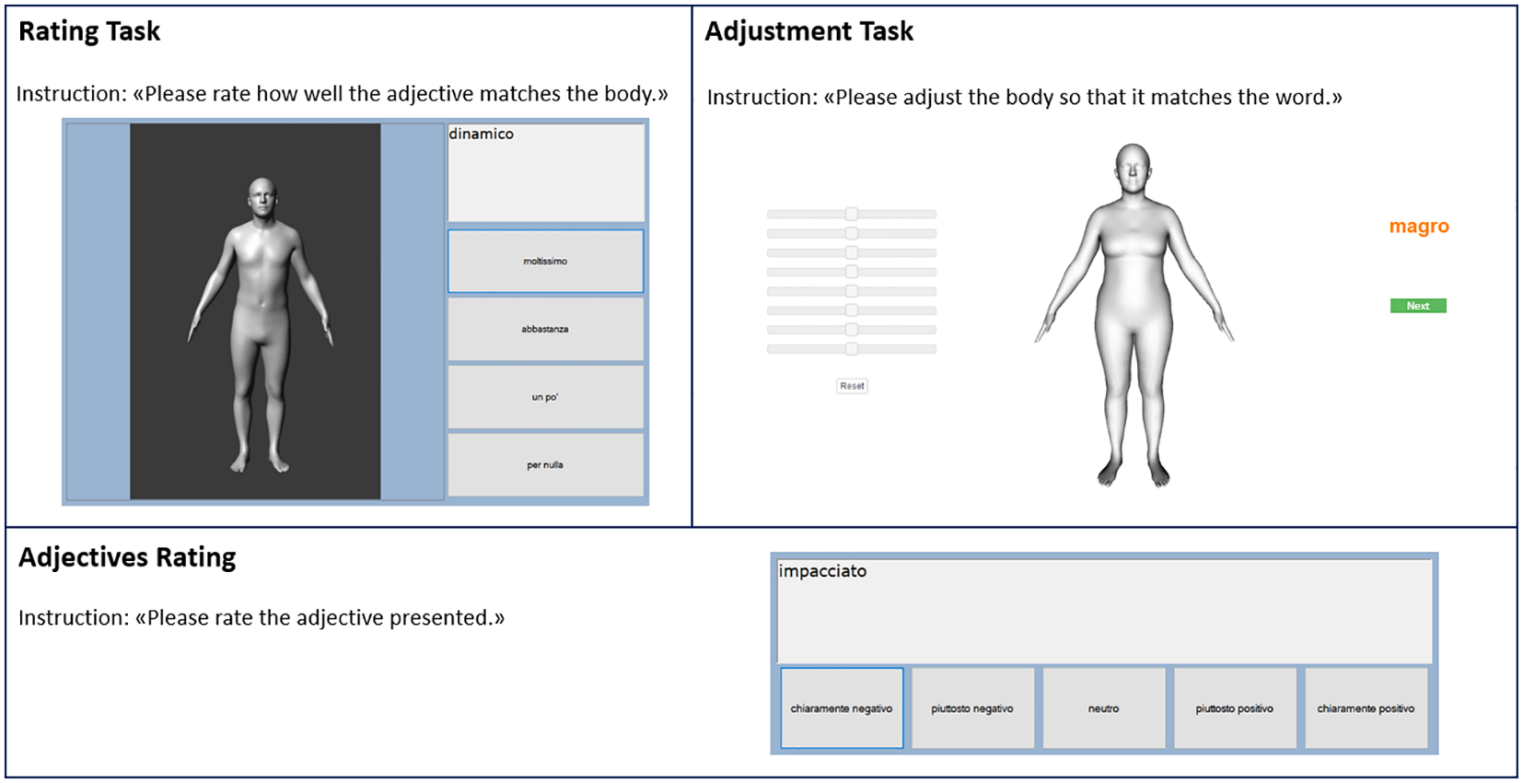
This figure depicts the two computerized tasks employed for assessment purposes. In a randomized sequence, participants were presented with 12 distinct body models alongside a set of 16 adjectives, both tailored to the gender of each participant.

Task 2: Adjustment task. The subsequent task, known as the “adjustment task”, tasked participants with generating distinctive prototypic bodies for each adjective. This was achieved by manipulating eight sliders positioned alongside the prototypic body images, which were not linked to specific body parts. We utilized eight sliders to apply a transformation based on the principal components of body shape, which are statistical descriptors that don’t directly correspond to specific body shape dimensions but seem to influence attributes like height, weight, leg length, or bust size. The initial shape of the displayed body was set to represent the average statistical model of human body shape (1.66 m, 69.5 kg for the female body), and all sliders were initially positioned in a neutral state. As the final step, participants were required to customize a body that closely matched their own, using the bars to modify the prototypic body, similar to the process employed for the adjectives. Following completion of the first two tasks, participants were prompted to rate each of the 16 adjectives on a 5-point scale, spanning from “clearly negative” to “clearly positive” (referred to as “adjective rating”). To mitigate potential sequence effects, the order of both adjectives and body images was randomized in the rating task. For a visual depiction of these tasks, please refer to **Figure 1**.

These computerized tasks have already been applied in different clinical populations (18, 19) and are available upon request.

### Statistical analysis

The normal distribution was evaluated using the Shapiro-Wilk test, and nonparametric statistics were applied when necessary. The t-tests were calculated to examine the differences between the clinical and control groups. For the evaluation of the adjusted own bodies in terms of their BMI, the Body Perception Index (BPI) was calculated according to the formula BPI = (estimated BMI/actual BMI) × 100 (18). Linear regression analyses were applied to evaluate the relationship between PHQ-9 scores and BPI, assessing the associations with depression and own body representation. Differences in the relationships between groups were evaluated with a General Linear Model (GLM) with an interaction between PHQ-9 and diagnoses. Additionally, t-test analyses were conducted to assess differences between self-representation associated with each adjective and the mental representation of bodies obtained through the adjustment task. To comprehensively assess implicit WB, we implemented a methodological approach inspired by the rating task. This involved the integration of valence ratings (ranging from -2 for clearly negative to 2 for clearly positive) and adjective match ratings (ranging from 0 for not at all to 4 for very much) with the body mass index of the displayed bodies. This integration was analogous to the computation of the Attitude variable in our study. The attitude score, indicated on a scale from -8 to +8, reflects the valence associated with each adjective across diverse body weights, signifying the potential presence of implicit WB. Negative values suggest an association with negatively valued adjectives, positive values with positively valued adjectives, and neutral values indicate a neutral valence. This nuanced approach, accounting for both valence and adjective-body matching, enhances our understanding of implicit WB. Additionally, we calculated the correlations between attitude and BMI adjusted in the rating task. The Mann-Whitney test assessed differences between groups across different BMI categories based on the WHO classification: underweight with a BMI < 18.5 kg/m²; normal weight with a BMI ≥ 18.5 and < 24.9 kg/m²; overweight with a BMI ≥ 25 and < 29.9 kg/m²; and obesity with a BMI ≥ 30 kg/m². For each category, a set of 3 figures was presented in the Rating task, and the means of the responses were used to evaluate the presence of differences between groups. The effect sizes were evaluated with Cohen’s d or η^2^. As recommended for exploratory studies, we did not perform multiple test adjustments (47), and the significance level was set at p <.05. All statistical analyses were performed with SPSS 25.0.

The sample size calculation was only an approximation due to the exploratory nature. A sample size similar to previous studies with the same tasks was chosen but in completely different clinical populations (people with anorexia nervosa and after bariatric surgery) (18, 19). We evaluated the sensitivity by examining statistical variations using an independent t-test with an uneven allocation ratio (n = 25 *vs*. n = 35). This demonstrated the ability to detect medium effect sizes of Cohen’s d = 0.775 with a calculated power of 0.90, using G*Power version 3.1 (48).

## Results

### Sample characteristics

**Table 1** summarizes the comparisons between both groups. The two groups did not differ in age, BMI, years of education, or ethnicity (all participants were White). The duration of MD was 4.5 years (range 2–8, SD 2.6). The MD group differed from the control group, showing higher scores in PHQ-9 (*p* = .001, *d* = 1.025) and DT (*p* = .001, *d* = .854) and lower scores at R-SES (*p* = .003, *d* = .817).

**Table 1:** Demographic characteristics of the included sample.

	MD n = 25	CP n = 35	t	*p*	*d*
Age, years	24.16 (3.61)	24.03 (2.37)	0.159	.874	0.043
BMI, kg/m^2^	22.26 (3.36)	21.72 (2.46)	0.716	.477	0.183
Education, years	16.68 (2.25)	17.49 (1.46)	-1.570	.125	0.427
Gender, female (%)	20 (80)	30 (86)	0.349*	.558	–
Bipolar disorder Major Depression	10 (40%) 15 (60%)	–	–	–	–
R-SES	14.83 (4.99)	18.80 (4.73)	-3.095	.003	0.817
PHQ-9	9.56 (4.64)	5.69 (2.65)	3.760	.001	1.025
DT	5.67 (5.42)	1.83 (3.32)	3.094	.001	0.854
BD	9.63 (7.85)	6.80 (5.56)	1.514	.113	0.416
BPI	110.83 (9.82)	104.06 (11.46)	2.391	.011	0.634
FPS	3.57 (0.61)	3.53 (0.34)	0.294	.746	0.081

MD, mood disorder; CP, comparison peers; BMI, body mass index; R-SES, Rosenberg self-esteem scale; PHQ-9, patient health questionnaire; DT, drive for thinness; BD, body dissatisfaction; BPI, body perception index; FPS, fat phobia scale; d, Cohen’s d. *: χ^2^.

### Weight bias

The fat phobia scale did not reveal differences between the two groups; therefore, there were no explicit differences in WB. Significant differences emerged only for one adjective in the adjustment task: insecure. For this word, the control group shaped a body with a higher BMI than the MD patients (MD BMI = 26.16 ± 7.77, CP BMI = 30.20 ± 6.22, *t* (56) = -2.187, *p* = .033, *d* = .574). See **Table 2** for details.

**Table 2:** Results from the adjustment task.

	MD	CP	*t*	*p*
Active	20.90 3.70	19.83 3.46	1.130	.263
Apple shaped	33.21 4.72	33.58 4.48	-0.162	.872
Attractive	20.58 2.76	20.25 2.05	0.531	.598
Clumsy	32.47 4.97	32.67 5.68	-1.058	.295
Determined	21.02 3.14	21.67 2.28	-0.860	.395
Feminine	21.50 3.50	21.57 2.80	-0.075	.941
Heavy set	29.27 3.98	30.42 4.76	-0.964	-1.151
Hourglass shaped	22.05 3.91	21.83 3.80	0.212	.833
Impulsive	23.73 5.44	25.04 6.37	-0.809	.422
Insecure	26.16 7.77	30.20 6.22	-2.187	.033
Lazy	32.47 4.97	32.67 5.78	-0.132	.896
Open minded	23.80 2.91	23.18 2.52	0.861	.393
Pear shaped	27.20 5.65	25.67 4.42	0.818	.417
Smart	23.68 2.81	22.70 2.43	1.400	0.167
Thin	17.66 2.78	17.83 2.77	-0.230	.819
Unfriendly	22.15 8.35	22.15 6.63	0.002	.999
Own body	24.12 5.72	22.71 3.86	1.134	.262

The table reported the means and standard deviations of the BMIs (kg/m^2^) resulting from the combination of the eight sliders in the task.

**Figure 2:**
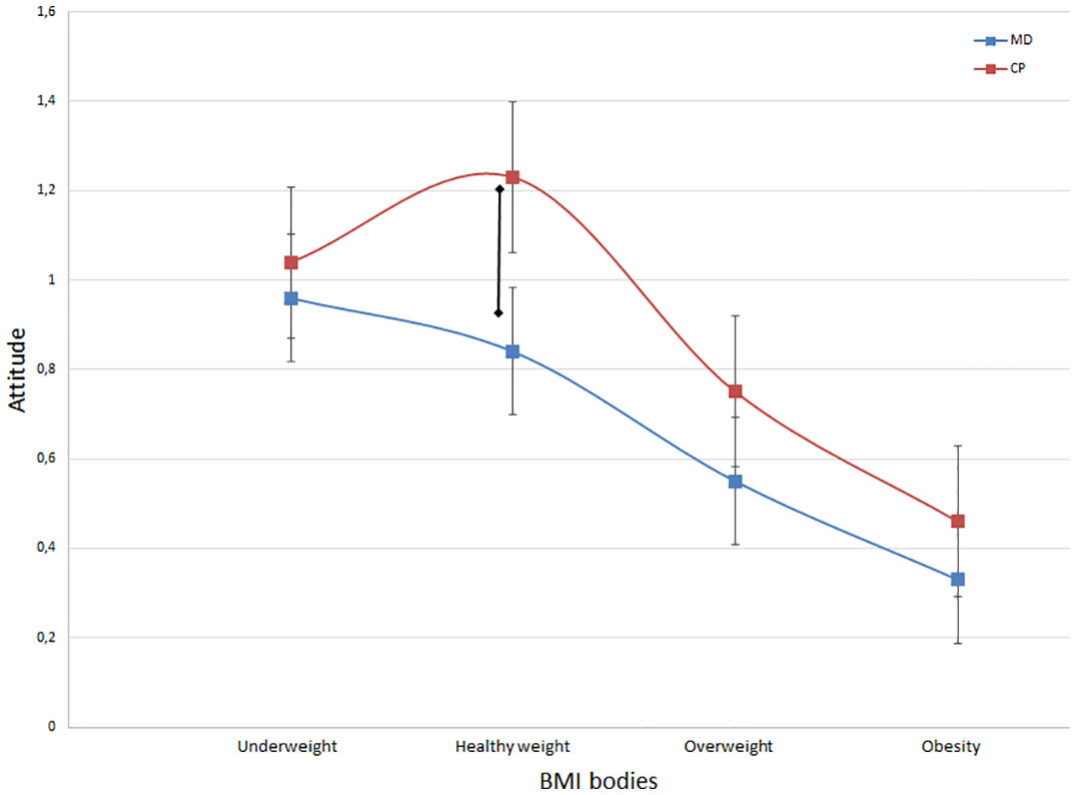
This graphical representation showcases the correlation between attitude and BMI categories for both CP and MD patients. Notably, a significant divergence between the two groups is evident within the healthy weight category (p = 0.012), characterized by the absence of the inverted U-shape curve observed in the patient group.

In the rating task, no WB emerged in the relationship between attitude and body BMI as shown in the task, with similar correlation coefficients between the two variables (MD *r* = -.072, *p* <.001; CP *r* = -.081, *p* <.001). However, looking at the distribution of evaluation of the body prototypes from the rating task, we can highlight a significant difference between subgroups in the weight range between 18.5 and 25 kg/m^2^ (BMI < 18.5 kg/m^2^: MD 0.96 ± 3.53, CP 1.04 ± 3.20, Z = -.764, *p* = .445; 18.5 < BMI < 25 kg/m^2^: MD 0.79 ± 3.57, CP 1.23 ± 3.19, Z = -2.452, *p* = .012, *η^2^* = .463; 25 < BMI < 30 kg/m^2^: MD 0.55 ± 3.54, CP 0.75 ± 3.18, Z = -1.105, *p* = .269; 30 kg/m^2^; BMI > 30 kg/m^2^: MD 0.33 ± 3.53, CP: 0.46 ± 3.09, Z = -.581, *p* = .561). These statistics show that MDs have a significantly less positive attitude than CPs toward weight considered healthier, suggesting that there is a higher implicit WB in MD patients than in CPs, as reported in **Figure 2**.

### Body image and self-representation

As shown in **Table 1**, the evaluation of body images showed the presence of a higher DT in the MD group than in the CP group (*p* =.001), although they had lower levels of body dissatisfaction. Regarding BPI, MD participants showed a higher overestimation of their body weight on the adjustment task (*p* = .012, *d* = 0.634), although both groups overestimated their sizes. Regarding the relationships between psychological constructs, linear regression analysis showed that the depression scores helped explain the results of the adjustment task results. Those with higher PHQ-9 were more likely to overestimate their size (*R^2^* = .313, *F* (1,22) = 10.028, *p* = .004, *B* = .571, *ES* = .180). This aspect differs from the results of the CP participants (*F* (1,33) = .313, *p* = .580). To confirm the differences between groups in this interaction, we conducted a GLM analysis, showing a significant interaction between PHQ-9 and diagnoses in the prediction of BPI scores (*F* (22) = 38.75, *p* = .015).

## Discussion

The primary objective of this study was to fully assess WB concerns among MD patients and to identify specific differentiators compared to their healthy counterparts. Understanding these distinctions could provide insight into the cognitive self-negative assessment that characterizes this population. Our findings support the core hypothesis, revealing that negative WB could be a component of the maladaptive cognitive patterns associated with depression.

When examining WB, our results revealed significant disparities in weight-based ratings between MD participants and healthy controls, particularly within the average BMI range. The patterns observed in our CP group are consistent with previous literature, showing an inverted U-shape trend in which individuals rated average-range weights (18.5 < BMI < 25.0 kg/m2) more favorably than underweight or overweight figures (49). On the contrary, our MD patients exhibited a distinct distribution in the evaluation of body shapes. Graphical representations indicated an implicit WB in both groups toward overweight and obese bodies, with no noticeable differences between them. However, MD patients did not manifest a more positive assessment of healthy weights (within the range of 18.5 to 25 kg/m2), as was evident in CP judgments. This absence of a favorable evaluation for healthy weights may help explain the higher Drive for Thinness scores and the greater overestimation of body weight reported by MD patients. The lack of a positive evaluation for healthy weights and shapes suggests a cognitive-affective distortion, indicating that these cognitive patterns may not be as all-encompassing and structured as those observed in eating disorders (18). However, they could still exert a detrimental impact on mood. Consequently, MD patients appear to view all aspects of themselves as inadequate, including their healthy weight. It should be noted that prior research has demonstrated an independent relationship between depressive symptoms and body weight dissatisfaction, regardless of BMI (50). Furthermore, studies have reported an association between uncorrected self-reported BMI and depression (51). In this context, implicit WB could play a role in psychopathology and requires further exploration.

Furthermore, our findings suggest a potential implicit cognitive disparity in the attraction of MD patients to thin-ideal bodies versus their aversion to non-thin bodies, which could contribute to internalized WB (15). Although no discernible differences in explicit WB emerged, it is possible that the structure of the FPS could limit disparities between implicit and explicit bias. However, the notion that negative self-evaluation may be more influenced by implicit assessments implies that future interventions should prioritize personalized implicit bias, particularly in the context of positive body image (52).

Finally, we observed no specific distinctions in body image concerns between MD patients and controls. This was a secondary aim in our study due to the limited evidence present in the literature. These findings align with the prevalent evidence that highlights the conceptual nature of body dissatisfaction in MD, which differs from the more perceptual distortion associated with eating disorders (53, 54). Our sample predominantly comprises young adults with relatively short disease duration, potentially contributing to a more accurate body evaluation (55). In particular, explicit body dissatisfaction was absent, a facet that has not been extensively evaluated in previous studies (26). Overall, our findings underscore the need for more research investigating body perception and depression between different age groups, as this area remains underrepresented in the current literature. It is also possible that the instruments applied might not be suitable for patients with MD due to their specific focus on eating disorders (56). This aspect should be evaluated in future research.

Furthermore, our results emphasized that drive to thinness is the predominant body-related concern among MD patients. This preoccupation may be related to an overestimation of body shape, as indicated by our findings. The DT construct could also be associated with the internalization of positive judgments surrounding low body weight, consistent with literature demonstrating such trends concerning average body representation (57). While DT is the primary concern associated with body shapes in the non-eating-disorder population, potentially reflecting the internalization of Western cultural ideals of thinness (15), it is noteworthy that DT also serves as a dysfunctional coping strategy for low self-esteem and depressive symptomatology (58, 59). From this point of view, our findings align with the body of evidence indicating that self-esteem, DT, and depression are interrelated constructs that significantly impact individuals’ psychological well-being, even among those without eating psychopathology or extreme weight conditions (i.e., extreme underweight or overweight) (60).

Several limitations intrinsic to our study’s experimental design warrant consideration. The most relevant limit of this study is the sample size of the groups included which limits the generalizability of the results. Given the exploratory nature of this study, our results should be validated with a larger sample size that includes a more diverse representation of genders (45). In addition, the assessment of psychological concerns surrounding the body was based on self-report scales. Future studies could employ various methodologies to evaluate both implicit and explicit WB within the MD population, potentially adopting a longitudinal approach to unravel the temporal and causal relationships between WB and depression. For example, implicit association tasks could be applied in this specific population to corroborate our results regarding the potential influence of judgments across different weight categories. Additionally, future body perception may need to encompass other cultural variables (e.g. sexual orientation) that could exert influence on clinical presentation (61). Lastly, the potential impact of depression deserves careful consideration. The existing literature on eating disorders has underscored the role of depression in WB and body shape assessment (62), which potentially signals a more pronounced influence among MD patients, which merits exploration through longitudinal investigations. Furthermore, future studies investigating potential distinctions between bipolar disorder and major depression could aid in stratifying these variances and identifying potential clinical applications for treatment.

## Conclusions

Despite inherent limitations, this study contributes to our understanding of self-evaluation patterns among individuals who are battling depressive symptoms. Our findings suggest the presence of implicit WB and an intriguing potential imbalance within the evaluation system. An aspect that our data contributes to the existing literature is the exploration of the connection between WB and depression in individuals with healthy weight ranges—an area that has received limited attention within the WB literature. The absence of a positive evaluation of body weight could potentially affect the implicit self-schema that individuals apply in their daily lives, thereby influencing the development or perpetuation of depressive symptoms. Furthermore, our findings underscore the multifaceted origin and widespread consequences of negative self-esteem schemes within the MD population. These findings suggest that targeted interventions aimed at addressing both self-evaluation and self-comparison processes could contribute to improving psychological and physical well-being.

## Data availability statement

The raw data supporting the conclusions of this article will be made available by the authors, without undue reservation.

## Ethics statement

The studies involving humans were approved by Padova Ethics Committees for Clinical Practice. The studies were conducted in accordance with the local legislation and institutional requirements. The participants provided their written informed consent to participate in this study.

## Author contributions

PM: Writing – original draft, Visualization, Software, Methodology, Investigation, Formal analysis, Data curation, Conceptualization. SB: Writing – original draft, Visualization, Validation, Supervision, Methodology, Formal analysis, Conceptualization. CP: Writing – review & editing, Investigation. TT: Writing – review & editing, Investigation. MQ-B: Validation, Supervision, Software, Writing – review & editing, Methodology, Conceptualization. MB: Validation, Supervision, Writing – review & editing, Project administration, Methodology, Conceptualization. KG: Writing – review & editing, Validation, Supervision, Conceptualization. ET: Writing – review & editing, Supervision, Methodology, Conceptualization. AF: Writing – review & editing, Supervision, Project administration, Methodology.
